# Receipt of Life-Sustaining Treatments for Taiwanese Pediatric Patients Who Died of Cancer in 2001 to 2010: A Retrospective Cohort Study

**DOI:** 10.1097/MD.0000000000003461

**Published:** 2016-04-22

**Authors:** Yen-Ni Hung, Tsang-Wu Liu, Dong-Tsamn Lin, Yueh-Chih Chen, Jen-Shi Chen, Siew Tzuh Tang

**Affiliations:** From the School of Gerontology Health Management and Master's Program in Long-Term Care, College of Nursing, Taipei Medical University (Y-NH); National Institute of Cancer Research, National Health Research Institutes (T-WL); Department of Pediatrics, National Taiwan University (D-TL); Department of Nursing, College of Medicine and Nursing, Hung Kuang University (Y-CC); Division of Hematology-Oncology, Chang Gung Memorial Hospital at Linkou and Chang Gung University College of Medicine (J-SC); and School of Nursing, Chang Gung University and Department of Nursing, Chang Gung Memorial Hospital at Kaohsiung (STT), Tao-Yuan, Taiwan, R.O.C.

## Abstract

Aggressive life-sustaining treatments have the potential to be continued beyond benefit, but have seldom been systematically/nationally explored in pediatric cancer patients. Furthermore, factors predisposing children dying of cancer to receive life-sustaining treatments at end of life (EOL) have never been investigated in a population-based study. This population-based study explored determinants of receiving life-sustaining treatments in pediatric cancer patients’ last month of life.

For this retrospective cohort study, we used administrative data on 1603 Taiwanese pediatric cancer patients who died in 2001 to 2010. Individual patient-level data were linked with encrypted identification numbers from the National Register of Deaths Database, Cancer Registration System database, National Health Insurance claims datasets, and Database of Medical Care Institutions Status. Life-sustaining treatments included intensive care unit (ICU) care, cardiopulmonary resuscitation (CPR), and mechanical ventilation. Associations of patient, physician, hospital, and regional factors with receiving ICU care, CPR, and mechanical ventilation in the last month of life were evaluated by multilevel generalized linear mixed models.

In their last month of life, 22.89%, 46.48%, and 61.45% of pediatric cancer patients received CPR, mechanical ventilation, and ICU care, respectively, with no significant decreasing trends from 2001 to 2010. Patients were more likely to receive all three identified life-sustaining treatments at EOL if they were diagnosed with a hematologic malignancy or a localized disease, died within 1 year of diagnosis, and received care from a pediatrician. Receipt of ICU care or mechanical ventilation increased with increasing EOL-care intensity of patients’ primary hospital, whereas use of mechanical ventilation decreased with increasing quartile of hospice beds in the patients’ primary hospital region.

Taiwanese pediatric cancer patients received aggressive life-sustaining treatments in the month before death. Healthcare policies and interventions should aim to help pediatricians treating at-risk pediatric cancer patients and hospitals with a tendency to provide aggressive EOL treatments to avoid the expense of life-sustaining treatments when chance of recovery is remote and to devote resources to care that produces the greatest benefits for children, parents, and society.

## INTRODUCTION

Pediatric cancer patients have greatly benefited from current treatments, but aggressive treatments might be continued beyond benefit and prolong suffering. This category includes life-sustaining treatments (LSTs) at the end of life (EOL), for example, intensive care unit (ICU) care,^1,2^ cardiopulmonary resuscitation (CPR),^3,4^ and mechanical ventilation.^5^

EOL care for adult cancer patients has become increasingly aggressive since the mid-1990s,^6,7^ but aggressive LSTs used at EOL have seldom been systematically/nationally explored in pediatric cancer patients.^8–11^ Furthermore, factors predisposing pediatric cancer patients to receive LSTs at EOL have never been investigated in a population-based study. Therefore, this study was undertaken to identify determinants of using LSTs in the last month of life for Taiwanese pediatric cancer patients who died in 2001 to 2010.

## METHODS

### Design and Sample

For this retrospective cohort study, digitized, individual patient-level data were linked with encrypted identification numbers from the National Register of Deaths Database (NRDD), Cancer Registration System (CRS) database, National Health Insurance (NHI) claims datasets, and Database of Medical Care Institutions Status (DMCIS). These databases’ completeness and accuracy were ensured by Taiwan's government. Identification of malignant neoplasms as cause of death in the NRDD is highly accurate (kappa = 0.94 with medical record reviews).^12^ The Taiwan Cancer Registry, a population-based cancer registry, stores all newly diagnosed malignancies, which must be reported by hospitals with >50 beds and providing cancer care. Most incident cancer cases (97.34%) were covered in the CRS, with 97.00% completeness and 91.11% accuracy.^13^

Taiwan's NHI provides universal coverage. Healthcare systems are reimbursed for services provided, and co-payment is waived for patients with malignancy. By 2010, 99.6% of 23 Taiwan's million residents were included in the NHI.^14^ NHI datasets were validated for accuracy in diagnostic coding, comorbidities, and healthcare-resource utilization, with clinical specialists routinely crosschecking medical records^15^ and measuring agreement between self-reported and NHI claims on healthcare utilization.^16^ Information quality on diagnoses, healthcare resource utilization, and patients’ EOL care has been verified.^15–18^ Information on hospital characteristics and healthcare resources for each hospital and region were obtained from the DMCIS.

Cancer was the top cause of non-accidental death in Taiwanese children, accounting for 6.9% to 9.3% of deaths in 2005 to 2010.^19^ The NRDD identified 1735 cancer deaths for individuals ≤18 years in 2001 to 2010. We deleted 132 cancer decedents from our analyses primarily because of missing data on disease characteristics (e.g., metastatic status) and their primary hospital's characteristics (e.g., hospital ownership). Therefore, our sample comprised of 1603 pediatric cancer decedents in 2001 to 2010. This study was approved by the Chang Gung Memorial Hospital Institutional Review Board, which waived the consent requirement. This study followed the STROBE guidelines.

### Measures

*Outcome variables* included receiving at least one of three LSTs (ICU care, CPR, and mechanical ventilation) in the last month of life to indicate aggressive EOL cancer care.^20,21^ ICU admission was ascertained from NHI inpatient hospital claims. Patients admitted to a traditional ICU were considered as using ICU care, but admissions to burn units or respiratory care units were excluded. CPR and mechanical ventilation were identified from inpatient claims by specific codes.

*Independent variables* included patient characteristics; primary physician's specialty; characteristics/healthcare resources; and EOL-care practice patterns both at the primary hospital and primary hospital's regional levels. These variables were chosen based on a conceptual framework for determining treatment intensity.^22^

Differences in outcome variables were examined across gender and age (from the NRDD). ICD-9 codes for primary and secondary diagnoses, excluding cancer-related codes, in inpatient and outpatient NHI claims in the year before death were used to identify comorbidities. The Deyo-Charlson comorbidity index was calculated by these ICD-9 codes and categorized as 0, 1, or ≥2 comorbid conditions.^23^ Diagnosis and date of diagnosis were identified from the CRS. At least 1 inpatient or 2 outpatient claims with ICD-9 codes 196.xx-199.xx at least 30 days apart^24^ in patients’ last year and stage IV indicated in CRS datasets since 2004 were used to identify metastatic status. Survival (i.e., the interval between dates of diagnosis and death) was categorized as 1 to 2, 3 to 6, 7 to 12, 13 to 24, and ≥25 months.

Primary physician specialty, retrieved from the physician-specialty code in NHI claims, was categorized into pediatricians (including pediatric hematologist/oncologists), medical oncologists/hematologists (primarily for adults), internists, surgeons, intensivists, and other. Primary physician was considered the physician who provided the most cancer care to an individual patient. In Taiwan, when a pediatric cancer patient's physical condition deteriorates and he/she may need ICU care, his/her primary physician consults an ICU physician to assess the necessity of patient transfer to the ICU for further management. When the primary physician and consultant ICU physician agree on patient admission to the ICU, the patient is transferred.

Characteristics/healthcare resources of the primary hospital, that is, where a patient had the most admissions during his/her last year, included hospital ownership and acute-care bed size from the DMCIS. Number of acute-care beds was grouped into the hospital's quartile ranking for bed size. Teaching status was not examined because 98.44% of subjects received cancer care in teaching hospitals.

Hospitals’ EOL-care practice patterns were captured by primary hospitals’ EOL-care intensity,^21^ which was assessed using a Medicare-spending measure, the End-of-Life Expenditure Index (EOL-EI).^25^ To construct hospital EOL-EI, we calculated individual-level age-sex-adjusted mean spending on inpatient, emergency-department, and outpatient services provided to cancer patients, regardless of age, in their last 6 months and aggregated spending to the primary hospital. Hospitals were grouped into quintiles of increasing EOL-care intensity.

Regional healthcare resources for patients’ primary hospital, that is, for the county/city where a specific hospital was located, were measured by the total number of acute-care and hospice beds in that specific region. These healthcare resources were divided into quartiles of beds/10,000 population from the DMCIS. Regional EOL-care practice patterns were indicated by EOL-care intensity and measured by regional EOL-EI.^25^ Each hospital's EOL-EI was calculated first as described above for primary hospitals and aggregated to each region for a cancer patient's primary hospital. Regional EOL-EI was categorized by quintile.

### Analysis

Multilevel generalized linear mixed modeling^26^ was used in a logit-link function by SAS GLIMMIX procedure to examine associations of each independent variable with receiving each LST at EOL. Considering the fact that individual patients clustered in the same hospital, each patient's primary hospital was used as a random effect, with independent variables used as fixed effects. The significance level was set at *P* ≤ 0.05 because of our study's exploratory design. Adjusted odds ratio (AOR) with 95% confidence interval (CI) was exponentiated from the regression parameter for each independent variable.

## RESULTS

The majority of subjects were male (Table 1) with a mean age (SD) of 9.93 (5.25) years (range = 1–18, median = 10.33). The top three cancer sites were blood/lymph (n = 726, 45.29%), brain (n = 439, 27.39%), and liver (n = 83, 5.18%). Over half the subjects had a non-metastatic disease and no comorbidities. Detailed patient characteristics are in Table 1.

**TABLE 1:**
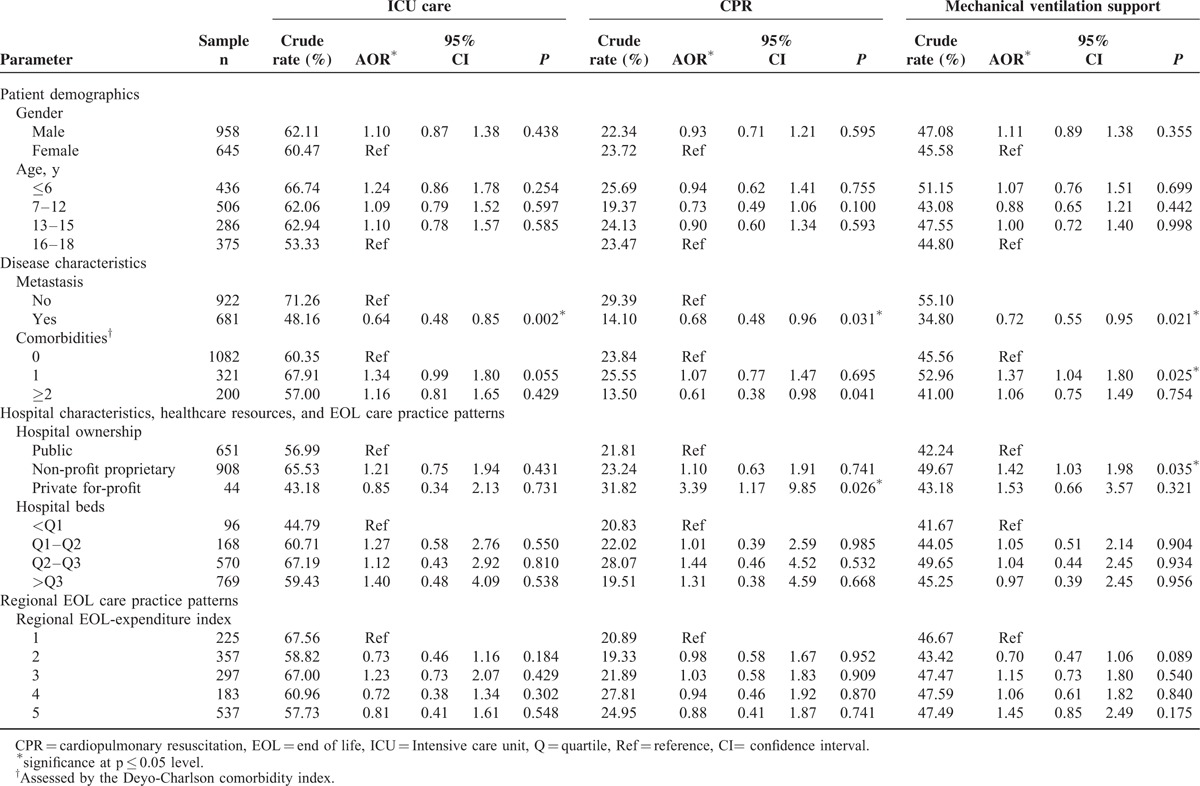
Determinants of Using ICU Care, CPR, and Mechanical Ventilation in 1603 Taiwanese Pediatric Cancer Decedents’ Last Month of Life

**FIGURE 1:**
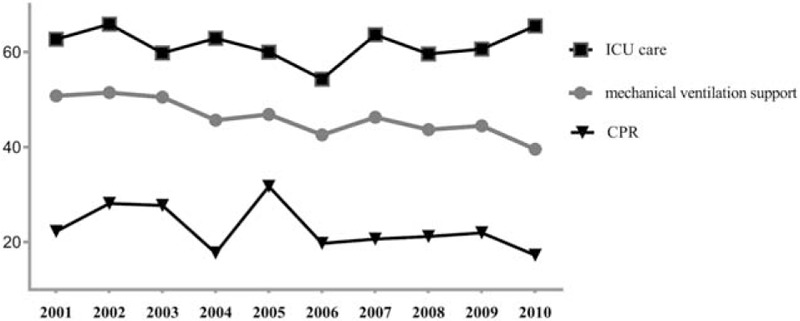
Trends in receiving ICU care, CPR, and mechanical ventilation in 1603 Taiwanese pediatric cancer decedents’ last month of life. CPR = cardiopulmonary resuscitation, ICU = intensive care unit.

Overall, 61.45% (range = 54.32–65.87%), 46.48% (range = 39.57–51.50%), and 22.89% (range = 17.27–31.72%) of Taiwanese pediatric cancer patients received ICU care, mechanical ventilation, and CPR in their last month, respectively (Figure 1). For all identified LSTs, trends from 2001 to 2010 did not decrease significantly in multivariate models.

**FIGURE 2:**
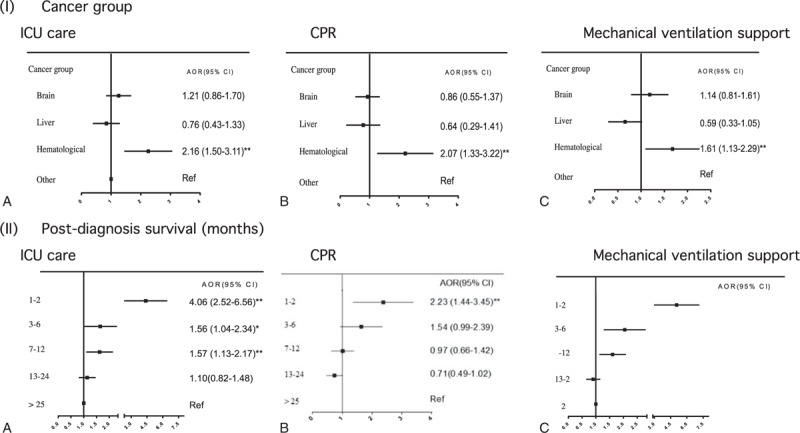
Associations of disease characteristics with receiving ICU care, CPR, and mechanical ventilation in 1603 Taiwanese pediatric cancer decedents’ last month of life. CPR = cardiopulmonary resuscitation, ICU = intensive care unit.

Taiwanese pediatric cancer patients’ propensity to receive LSTs in their last month was not associated with their demographic characteristics (Table 1). This propensity was significantly higher for children with a hematologic malignancy (Figure 2I) and a localized disease (Table 1) than for children with other cancer diagnoses and metastatic disease. Patients who died within 1 year of diagnosis were significantly more likely to receive all identified LSTs in their last month than those who died ≥13 months after diagnosis (Figure 2II). Moreover, patients with 1 comorbidity were significantly more likely to receive mechanical ventilation, whereas those with ≥2 comorbidities were less likely to be resuscitated in their last month than those without any comorbidity (Table 1).

**FIGURE 3:**
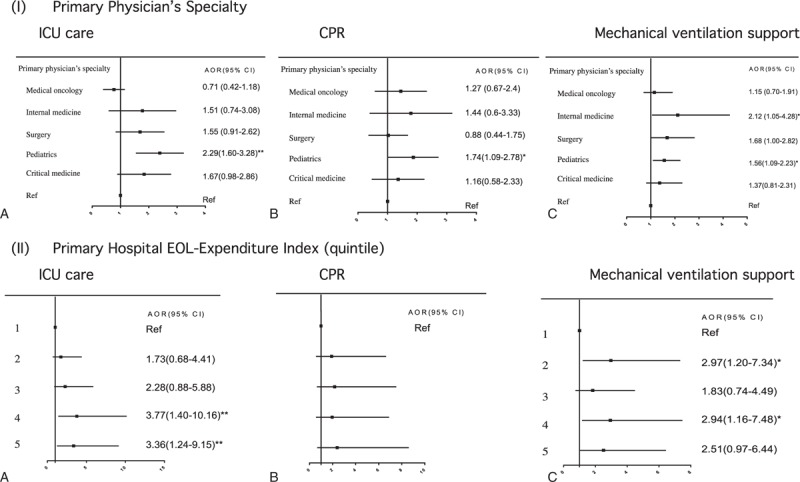
Associations of primary physician's specialty and primary hospital EOL-Expenditure Index with receiving ICU care, CPR, and mechanical ventilation in 1603 Taiwanese pediatric cancer decedents’ last month of life. CPR = cardiopulmonary resuscitation, ICU = intensive care unit.

Patients whose primary physician was a pediatrician were significantly more likely than patients cared for by other physician specialists to receive all three LSTs (Figure 3I) in the last month. The propensity to receive LSTs in a child's last month was not associated with the primary hospital's healthcare resources (bed size) (Table 1). However, patients were more likely to be resuscitated and receive mechanical ventilation support in their last month, respectively, if they were cared for in a private-for-profit or non-profit proprietary than in a public hospital. Furthermore, children cared for in a primary hospital with a higher intensity EOL-care practice had a greater propensity to receive ICU care and mechanical ventilation support (Figure 3II).

**FIGURE 4:**
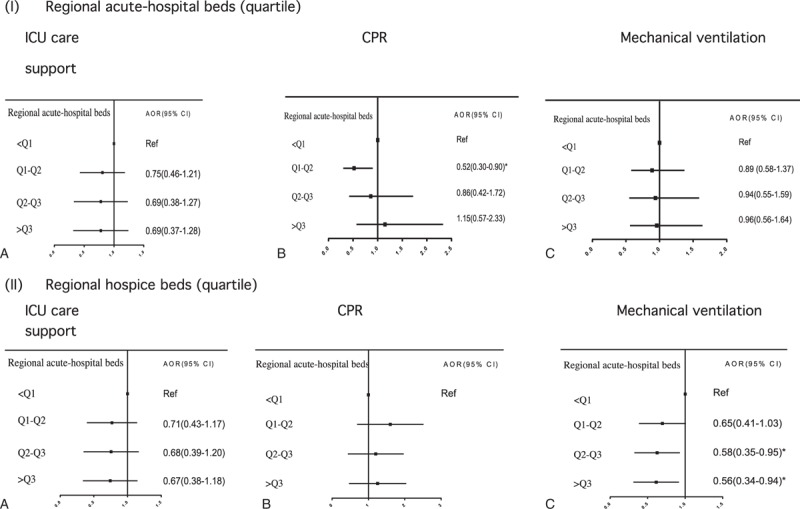
Associations of regional healthcare resources with receiving ICU care, CPR, and mechanical ventilation in 1603 Taiwanese pediatric cancer decedents’ last month of life. CPR = cardiopulmonary resuscitation, ICU = intensive care unit.

The likelihood of being resuscitated and receiving mechanical ventilation support in the last month decreased with more acute-care hospital and hospice beds in the region of patients’ primary hospitals, reaching significance for regions with the second and the two largest quartiles of acute-care hospital and hospice beds, respectively (Figure 4). However, regional EOL-care practice patterns (indicated by regional EOL-EI) were not associated with receiving any identified LST.

## DISCUSSION

Taiwanese pediatric cancer patients received aggressive LSTs at EOL, as shown by 61.45% receiving ICU care, 46.48% receiving mechanical ventilation, and 22.89% receiving CPR in their last month. These figures are substantially higher than previous reports. ICU care was received by 22% to 38% of the United States^8,9^ and 41.4% of Korean^11^ pediatric cancer patients. Intubation or mechanical ventilation before death was provided to 11% to 21% of US pediatric cancer patients.^8,9,27^ CPR was attempted in 7.0% to 12.0%, 8.3%, 16.2%, and 25% of pediatric cancer patients from the United States^8,27,28^, Australia,^29^ Korea,^11^ and Japan,^30^ respectively.

The substantially higher propensity for our sample to receive LSTs in their last month may reflect the Chinese/Taiwanese cultural attitude that dying young defies the cultural value of filial piety. Therefore, Taiwanese parents tend to insist on “resuscitating until the last second” to save their child's life, consistent with Taiwanese pediatric patients’ higher CPR incidence than reported from Australia and Canada.^31^ However, the United States parents are also highly willing to have their child undergo major adverse effects for a small treatment benefit to avoid death.^32^ Therefore, our nation-wide results may provide insights to researchers from other countries considering the systematic study of trends in aggressive LSTs for pediatric cancer patients at EOL.

We found no significant, consistent, decreasing trends toward less use of all identified LSTs from 2001 to 2010, echoing trends in other countries. Receiving ICU care^11^ and mechanical ventilation support^9,11^ did not change significantly for Korean and both Korean and the United States pediatric patients, respectively, over time. However, the proportions of Korean pediatric cancer patients receiving CPR decreased significantly from 2007 to 2010.^11^ Similarly, using ICU care for pediatric patients decreased over time in the United States^9^ and Germany.^33^ Our finding that receiving LSTs did not decrease in Taiwanese children's last month highlights the urgent need to integrate palliative care into pediatric cancer care^33^ as a standard of EOL care. Less than 7.2% of pediatric cancer patients received hospice care before they died in 2001 to 2006.^10^ Although the number of hospice programs in Taiwan increased substantially from 2004 to 2010, pediatric hospice care is non-existent to sparse. Indeed, hospice home care increased from 49 to 74, inpatient hospice units increased from 27 to 47, and hospital-based palliative care teams increased from 8 to 71 from 2004 to 2010.^34^ However, no hospice (either inpatient unit or home care) in Taiwan is dedicated to pediatric patients, and few if any hospice care teams exist to meet the palliative care needs of pediatric cancer patients. Integrating palliative care into standard oncology care may gradually counteract the inclination of Taiwanese parents^31^ and society to avoid accepting that a child is at EOL by promoting comfort and quality of life instead of prolonging terminally ill children's life.

Our pediatric patients were significantly more likely to receive LSTs in their last month, if they were diagnosed with a hematologic malignancy or localized disease. These characteristics may represent children with an early stage disease associated with a good survival potential. Children/adolescents diagnosed with leukemia/lymphoma are more likely than those with brain or other solid tumors to undergo CPR,^28,30^ receive LSTs,^8,27^ and die in an ICU.^27^ The high survival rates of children with hematologic malignancies or localized diseases may decrease both families’ and physicians’ likelihood of recognizing the child as being beyond cure and letting “nature take its course” as reasons for limiting curative therapies and LSTs to fight disease progression at EOL.^35,36^

Furthermore, children with newly diagnosed disease (i.e., dying within 1 year of diagnosis) tend to receive curative treatment,^8,37^ which may hinder healthcare professionals from initiating EOL-care discussions with parents. In this situation, little time is left to prepare for death or consider EOL-care options, including using LSTs when the child's life is in danger from treatment-related complications.^27^ Indeed, not planning for a child's death has been associated with more CPR attempts within 24 hours of death and more pediatric cancer patients dying in an ICU.^37,38^

Our pediatric subjects were significantly more likely to receive LSTs in their last month if they received care from pediatricians than from other specialists. Pediatric oncologists often feel insufficiently trained, inexperienced, and uncomfortable in EOL care.^39,40^ Taiwanese pediatricians may suffer more in providing EOL care than western pediatric oncologists because palliative care is a relatively young field in Taiwan and few children dying of cancer receive hospice care.^10^ Furthermore, longer relationships between oncologists and their adult patients at EOL have been associated with more rescue care^41^ and a greater predisposition to be influenced by family appeal.^42^ Taiwanese pediatricians with long-term, trusting relationships with parents of pediatric cancer patients but poorly trained in palliative care may empathize with parents’ struggle to let go by ordering LSTs.^43^ To develop interventions that optimize Taiwanese pediatricians’ care for children dying of cancer, research is urgently needed on pediatricians’ reasons and motivations for aggressive EOL care.

Children receiving care in non-public hospitals were significantly more likely to be resuscitated or receive mechanical ventilation support in their last month, as reported for adult cancer patients.^44^ Non-public hospitals seek to maximize profits, which may predispose affiliated physicians to treat children more aggressively at EOL. Furthermore, Taiwanese physicians affiliated with non-public hospitals are more likely to lack palliative/hospice education and experiences necessary to appropriately provide EOL care to dying children because the hospice movement in Taiwan was initiated largely by healthcare professionals at public hospitals, where hospice has been more readily integrated into cancer care.

We report the novel result that the propensity to receive LSTs in the last month of children's life was determined by their primary hospital's EOL-care practice patterns, outweighing the influence of traditional hospital healthcare resources—acute-care bed size.^44^ EOL-care intensity has been associated with aggressive EOL care for adult patients in the United States at the regional level,^45^ but has never been explored at the primary hospital level. However, the impact of regional variation in EOL-care intensity on healthcare spending and service utilization was questioned when the primary source of geographic variation in healthcare spending was identified as patients’ underlying health status.^46^ In contrast, EOL-care intensity is considered a “real” indicator of how aggressively a hospital treats terminally/critically ill patients.^21^ We found that receiving care at a hospital with greater EOL-care intensity predisposed pediatric cancer patients to receive ICU care and mechanical ventilation support at EOL.

The propensity to use mechanical ventilation support in Taiwanese pediatric cancer patients’ last month varied by regional hospice bed supply. Our results are consistent with greater local availability of hospice being associated with less aggressive treatment (including ICU care) in US adult cancer patients’ last month.^47^ Inpatient palliative care programs have been shown to facilitate the transit of critically ill/dying patients out of ICUs.^48^ By the same token, hospice philosophy may diffuse into pediatric oncology care in areas with abundant hospice beds to impact EOL-care practice. However, our finding that the likelihood of receiving CPR was lower in regions with the second highest quartile of acute-care hospital beds than in the largest quartile warrants further investigation.

The strength of our study lies in its population-based sample encompassing all ages and cancer groups of Taiwanese pediatric cancer patients as well as a broad range of potential predictors of receiving LSTs in patients’ last month.^22^ However, our observational design allows only conclusions based on associations; causality cannot be inferred. We recognize that the statistical significance for some variables may have relatively little clinical significance and the potential for inflated Type I errors due to multiple tests performed in multivariate models. Observational studies can never exclude possible residual confounders, such as children's and their parents’ EOL-care preferences and differences in primary physicians’ evaluation of the appropriateness of using LSTs at EOL. The appropriateness of receiving LSTs depends on accurately defined populations of patients dying from complications of curative cancer treatment or of cancer.^49^ We could not ascertain this distinction from the NHI claims database because important clinical features regarding patients’ disease stage and needs are not included.

## CONCLUSIONS

Taiwanese pediatric cancer patients received aggressive LSTs in their last month with no significant decreasing trends over time. When these patients’ physical condition deteriorates, a systematic team approach is desirable to thoroughly evaluate each patient's disease progression as well as the effectiveness of continuing anti-cancer treatments and further LSTs. The team should include pediatricians, palliative experts, oncology nurses, psychologists, and social workers with input from the patient (if appropriate) and his/her parents regarding future care goals. Common care goals supported by all stakeholders will not only benefit patients, but also lift pediatricians’ burden of decision-making and may bridge gaps in their EOL-care training to achieve appropriate care for terminal pediatric cancer patients. Clinical interventions should also be developed to facilitate parents’ early recognition that receiving LSTs for at-risk children at EOL does not realistically improve their chance for cure.^1–5^ Health policies should target hospitals with a tendency to provide aggressive EOL treatments to ensure that the expense of LSTs is avoided when chance of recovery is remote and that resources (i.e., increasing regional hospice beds and palliative care training for pediatricians) are devoted to care that produces the greatest benefits for children, parents, and society.
